# Pancreatic pseudocyst with pancreatolithiasis and intracystic hemorrhage treated with distal pancreatectomy: a case report

**DOI:** 10.4076/1757-1626-2-8693

**Published:** 2009-08-24

**Authors:** Masato Maeda, Ryota Nomura, Toshiaki Moriki, Tadashi Miyashita

**Affiliations:** 1 Department of Surgery, Shizuoka City Shizuoka Hospital10-93 Ohtemachi Aoi-ku, Shizuoka 420-8630Japan; 2 Department of Pathology, Shizuoka City Shizuoka Hospital10-93 Ohtemachi Aoi-ku, Shizuoka 420-8630Japan

## Abstract

**Introduction:**

Hemorrhage from pancreatic pseudocyst is one of the serious complications of chronic pancreatitis. We experienced intracystic hemorrhage from a huge pancreatic pseudocyst and successfully treated it with distal pancreatectomy.

**Case presentation:**

A 65-year-old-man with a history of alcohol abuse was admitted to our hospital for abdominal pain and was diagnosed as having chronic pancreatitis with pancreatolithiasis and pseudocyst in the pancreatic tail. The pancreatic pseudocyst increased in size gradually for 4 month observation period. For intracystic hemorrhage we performed an urgent distal pancreatectomy with splenectomy. Postoperative course was good and the elevated serum amylase level decreased to the normal range.

**Conclusion:**

Prolonged observation resulted in intracystic hemorrhage. Drainage or surgery in adequate time is important for the management of pancreatic pseudocysts to prevent complications.

## Introduction

Psuedocyst of the pancreas is a common and painful complication of chronic pancreatitis and complicates about 20% to 50% of patients after chronic pancreatitis [1]. Several complications of pancreatic pseudocysts have been reported, e.g., rupture, infection, biliary obstruction and hemorrhage. Hemorrhagic complications are expected in 6-31% of patients with pancreatic pseudocysts and can be life-threatening condition [2]. This article reports a case of intracystic hemorrhage from a huge pancreatic pseudocyst successfully treated with an urgent distal pancreatectomy.

## Case presentation

**Figure 1.**
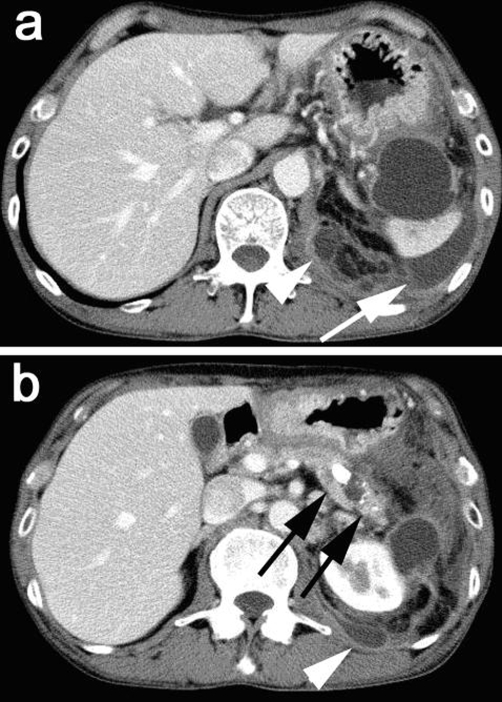
An abdominal CT scan at the 1st consultation showed many stones in the main pancreatic duct (**black arrows in b**) and a cystic lesion, 4 cm diameter, located in the pancreatic tail and surrounded by the stomach, the spleen and the left kidney. The cystic lesion extended to the dorsal side of the left kidney (**white arrowhead in b**) and there was small amount of fluid collection around the spleen, which was considered to be caused by acute inflammation on chronic pancreatitis (**white arrow in a**).

A 65-year-old Japanese man was admitted to our hospital in April 2007 for abdominal pain after drinking alcohol. The patient details are as follows: Occupation: company employee; Ethnicity: Japanese; Weight: 45 kg; Height: 157 cm; Medical history: no appreciable disease ; Family history: unremarkable ; Habits: 900 ml of Japanese Sake (corresponding to 1,200 ml of wine) per day for 45 years and 20 cigarettes per day for 45 years. Abdominal examination carried out on the 1st consultation showed mild tenderness on the epigastric region without significant rebound and a palpable mass. The reminder of his examination was unremarkable. Laboratory tests showed the following values: amylase, 811 IU/L (reference level, 40-120); lipase, 732 IU/L (reference level, 17-57); Elastase-1, 2811 ng/ml (reference level 72-432); C-reactive protein, 3.24 (reference level 0.00-0.30). Values of other blood chemistry and hematology were unremarkable. An abdominal computed tomography (CT) scan showed many stones in the main pancreatic duct and a cystic lesion, 4 cm diameter, located in the pancreatic tail, that was diagnosed as pancreatic pseudocyst with pancreatolithiasis accompanied with chronic pancreatitis (Figure 1).

**Figure 2.**
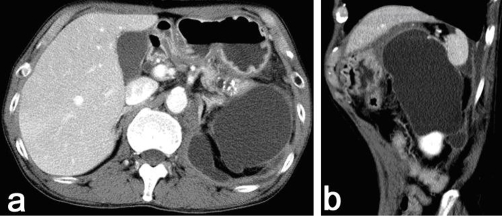
After 4 months conservative treatment CT scan demonstrated the cyst increased in size to 7 cm diameter on horizontal section **(a)** and 11 cm in the cranio-caudal direction **(b)**.

Initially the patient was treated conservatively but 6 weeks later abdominal pain had become severe and the cyst increased in size upto 6 cm diameter on CT scan. We recommended surgery, but the patient refused to undergo an operation and hoped medical management. Treatment using gabexate mesilate diminished his symptom but did not decrease the serum amylase level. 4 months after his first consultation abdominal pain became severe again and the cyst increased in size (Figure 2).

**Figure 3.**
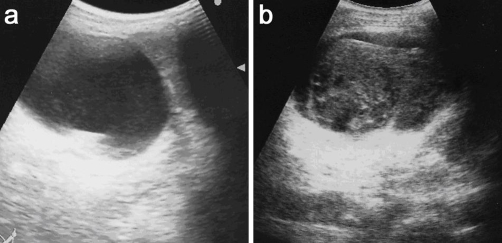
Ultrasonography examination revealed heterogeneous mass in the cyst **(b)**, which had not been found 3 days before **(a)**.

Once his symptom was relieved, after several days he complained a sudden and more severe left upper quadrant pain. Ultrasonography examination revealed heterogeneous mass in the cyst, which suggested intracystic hemorrhage (Figure 3).

**Figure 4.**
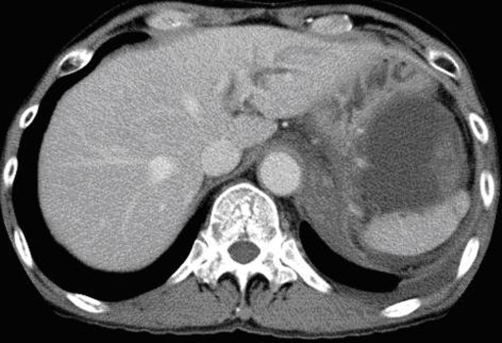
CT scan demonstrated high density structure in the cyst.

CT scan also demonstrated high density structure in the cyst, which had not been found out on the previous CT scan, but showed no aneurysm or extravasation apparently (Figure 4).

**Figure 5.**
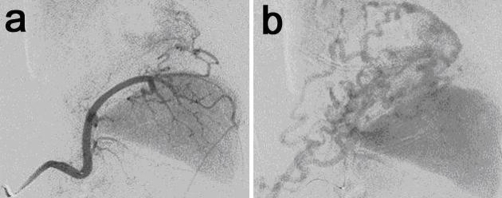
Angiography examination from the splenic artery demonstrated no aneurysm or extravasation **(a)** and showed a collateral formation in the greater curvature side of the stomach instead of the splenic vein in the late phase **(b)**.

Although the patient showed no sighs of shock, we diagnosed it as an intracystic hemorrhage and immediately selective angiography examination (SAG) was performed. SAG from celiac trunk or splenic artery demonstrated no aneurysm or extravasation (Figure 5a) but showed complete obstruction of the splenic vein and a collateral formation in the greater curvature side of the stomach (Figure 5b).

**Figure 6.**
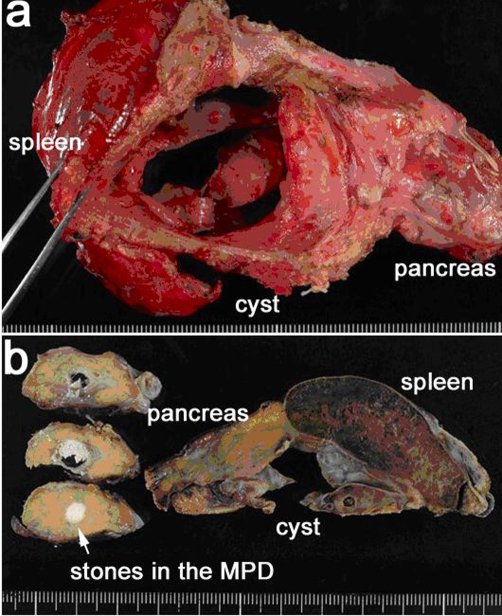
Raw resected specimen was observed from the dorsal side **(a)**. Sagittal section (**left side of b**) and coronal section (**right side of b**) of formalin fixed specimen. MPD; main pancreatic duct.

Then we performed an urgent operation, which disclosed that the cyst of diameter 7 cm size involving the pancreatic tail and the spleen adhered firmly to the stomach and the colon, and the lower part of the cyst wall was united with a circumference fascia of the left kidney. A distal pancreatectomy with splenectomy was performed to remove all the stones in the pancreas and the cyst wall was removed partially to preserve the stomach, the colon and the left kidney (Figure 6).

The content of the cyst was dark reddish-brown bloody liquid and blood coagula. The value of amylase and CA19-9 of the liquid was 48,520 U/L and 7,880 U/ml, respectively. Histopathological finding revealed that no epithelium was observed in the inner lining and fibrosis and infiltration of inflammatory cells in the surrounding pancreatic tissue with atrophy was observed, then it was diagnosed as a chronic pancreatitis with a pseudocyst without any malignant portion. Postoperative course was uneventful and the serum amylase value decreased to the normal range, then the patient was discharged on postoperative day 18.

## Discussion

A pancreatic pseudocyst is a localized fluid collection within or adjacent to the pancreas, enclosed by a nonepithelialized wall. As acute pseudocysts are known to resolve spontaneously in considerable frequency, expectant management for at least 4 to 6 weeks should precede surgery or intervention [3]. In contrast chronic pseudocysts rarely regress if they are larger than 4 to 6 cm in diameter [2]. As the pancreatic pseudocyst in our case, which was accompanied with an alcoholic chronic pancreatitis, was referred to a chronic pseudocyst, spontaneous regression was hardly expected. In addition the stones in the main pancreatic duct were considered to disturb the flow of the pancreatic juice toward the duodenum and allow the cyst to become gradually greater. Acute hemorrhage from a pseudocyst is one of the most serious complications of chronic pancreatitis. The sudden onset of an abdominal pain was considered by rapidly increased intracystic pressure due to acute hemorrhage. The progressive enlargement of the pseudocyst induces necrotizing vessels because of the added pressure on the vessel walls and from the action of proteolytic enzymes. This leads to the erosion of the vessel walls, resulting in intracystic hemorrhage [4]. Another important cause of intracystic hemorrhage is the rupture of the pseudoaneurysm, which often results in massive bleeding to cause shock status and is usually treated by emergency transarterial embolization (TAE) or operation [5]. As no pseudoaneurysm or extravasation was detected on the angiography examination, we performed urgent operation without TAE.

The accepted opinion about the timing of treatment for pseudocysts has been as follows: (i) a pseudocyst that occurs after an episode of alcohol-related pancreatitis has to be observed for 4 to 6 weeks. (ii) After 6 weeks observation should continue if the size of the cyst is less than 6 cm and the patient is asymptomatic or if there is decrease in size. (iii) Therapy is indicated if the patient is symptomatic or if the cyst size is more than 6 cm, the cyst is increasing in size, the cyst is infected, or there is a suspicion of malignancy. (iv) Observation is unnecessary, and immediate drainage is safe in cysts that have a mature wall or in those arising in chronic pancreatitis [6]. Percutaneous drainage of cysts is the least invasive method, however it is not the procedure of the choice in the presence of a drainage disturbance in the main pancreatic duct like our case, because of the risk of a permanent external fistula [6]. Percutaneous and trans gastric puncture of pancreatic cysts to make cystogastrostomy guided by ultrasonogtaphy and gastroscopy, has the advantage of establishing an internal fistula between the cyst and the stomach and preventing a pancreatic cutaneous fistula [7]. Endoscopic drainage is also less invasive and makes internal fistula to achieve a low recurrence rate As operative methods cystgastrostomy or cystjejunostomy to make internal drainage is often performed and recently it may be done laparoscopically [8]. However we have to pay attention to the fact that massive hemorrhage can occur not only in untreated pseudocysts but also following internal drainage [9]. Although resection of cysts is usually accompanied with pancreatic resection and is more invasive method, the episode of intracystic hemorrhage made the choice of anastomosis of the cyst to the alimentary tract risky for recurrent hemorrhage and a removal of the pancreatic stones as well as the cyst by pancreatic resection provided definitive control [10].

## Conclusion

Chronic pancreatic pseudocyst rarely regress if they are larger than 4 to 6 cm in diameter. As the patient had initially refused an operation, conservative treatment was performed for a too long period in our case, that allowed the cyst growing and hemorrhagic complication. Drainage or surgery in adequate time is important for the management of pancreatic pseudocysts to prevent complications.

## Abbreviations

CA19-9: carbohydrate antigen 19-9
CT: computed tomography
SAG: selective angiography
TAE: transarterial embolization
